# Environmental Pollutants PM2.5, PM10, Nitrogen Dioxide (NO_2_), and Ozone (O_3_) Association with the Incidence of Monkeypox Cases in European Countries

**DOI:** 10.1155/2023/9075358

**Published:** 2023-01-13

**Authors:** Sultan Ayoub Meo, Sara A. Alqahtani, Anusha Sultan Meo, Joud Mohammed Alkhalifah, Thamir Al-Khlaiwi, David C. Klonoff

**Affiliations:** ^1^Department of Physiology, College of Medicine, King Saud University, Riyadh 11461, Saudi Arabia; ^2^College of Medicine, King Saud University, Riyadh 11461, Saudi Arabia; ^3^U.C. San Francisco and Diabetes Research Institute, Mills-Peninsula Medical Center, San Mateo, CA, USA

## Abstract

**Background:**

Monkeypox, also known as monkeypox disease, is caused by the monkeypox virus (MPXV), which is a zoonotic infection. The swift spread of human monkeypox cases has caused an alarming situation worldwide. This novel study aimed to investigate the association of particulate matter air pollutants PM2.5, PM10, Nitrogen dioxide (NO_2_), and Ozone (O_3_) on the incidence of monkeypox cases from May 1, 2022, to July 15, 2022.

**Methods:**

The data on air pollutants PM2.5, PM10, NO_2_, and O_3_ and monkeypox cases were recorded from the date of occurrence of the first case of monkeypox in the United Kingdom, Spain, France, Germany, Italy, the Netherlands, Switzerland, and Portugal from May 1, 2022, to July 15, 2022. The daily concentrations of PM2.5, PM10, NO_2_, and O_3_ were recorded from the metrological website “Air Quality Index-AQI,” and daily human monkeypox cases were recorded from the official website of “Our World in Data.” The mean values along with simple, multiple, and Spearman Rho correlations were performed to investigate the relationship and strength of association between the concentrations of air pollutants and cases of monkeypox.

**Results:**

The environmental pollutants PM2.5, PM10, NO_2_, and O_3_ were positively associated with monkeypox cases in the United Kingdom, Spain, France, Germany, Italy, the Netherlands, Switzerland, and Portugal. The analysis further revealed that for each 10-unit increase in PM2.5, PM10, and NO_2_, levels, the number of monkeypox cases was significantly augmented by 29.6%, 9.7%, 13%, and 80.6%, respectively.

**Conclusions:**

Environmental pollutants PM2.5, PM10, NO_2_, and O_3_ have been positively linked to the number of daily monkeypox cases in European countries. Environmental pollution is a risk factor for the increasing incidence of monkeypox daily cases. The regional and international authorities must implement policies to curtail air pollution to combat the cases of monkeypox in European countries and worldwide.

## 1. Introduction

The human monkeypox disease is caused by the monkeypox virus (MPXV), which is a zoonotic infection most commonly found in African countries. The MPXV belongs to the “genus *Orthopoxvirus,* subfamily *Chordopoxvirinae* and family *poxviridae*” [1, 2]. The monkeypox virus was found for the first time in 1958 after the occurrence of a pox-like ailment in monkeys, which were housed in the research institute in Copenhagen, Denmark. Hence the disease acquired the name monkeypox [3]. About 12 years later, in September 1970, the MPXV was identified in humans in the Democratic Republic of the Congo [4–6]. Human monkeypox disease was later transmitted outside the endemic African countries to nonendemic nations, including the United States of America in 2003 [7].

This year, from early May 2022, the monkeypox virus swiftly spread from nonendemic to endemic regions. On August 19, 2022, the disease had involved 94 countries worldwide and had infected 41,358 people: 387 cases from endemic 07 African countries and 40,971 cases from 87 nonendemic countries in Europe, North and South America, Australia, and Asia [8]. On July 23, 2022, the World Health Organization (WHO) declared the monkeypox outbreak to be a global public health emergency of worldwide concern [9]. The swift spread of monkeypox cases has caused an alarming situation globally [10]. The possible routes of transmission of MPXV are animal-to-human and human-to-human. Respiratory droplets, direct or indirect contact with body fluids, skin lesions of an infected person, and contaminated surfaces in a patient's environment have been associated with interhuman transmission [2, 11–13].

The world is witnessing that viral infections have a transmission linkage with environmental conditions. Since December 2019, the transmission of Severe Acute Respiratory Syndrome Coronavirus 2 (SARS-CoV-2) has been highly associated with exposure to environmental pollutants [14, 15]. The science community has been trying to identify a possible linkage between the spread of human monkeypox cases and its association with environmental pollution. This study is aimed at investigating the association between environmental pollutants PM2.5, PM10, CO, O_3_, and NO_2_ and daily cases of monkeypox disease in European countries.

## 2. Materials and Methods

**Figure 1:**
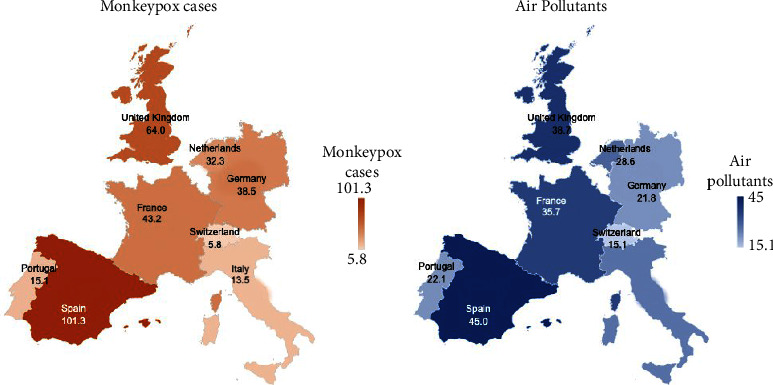
Mean levels of environmental pollutants PM2.5, PM10, NO_2_, and O_3_, and monkeypox cases in European countries.

The present study was conducted in the “Department of Physiology, College of Medicine, King Saud University, Riyadh, Saudi Arabia.” This study investigated the association of four environmental pollutants, namely particulate matter PM2.5, PM10, NO_2_, and O_3_, in eight countries in Europe and their association with monkeypox cases. The eight selected European countries were the United Kingdom, Spain, France, Germany, Italy, the Netherlands, Switzerland, and Portugal (Figure 1). The daily number of cases and concentrations of environmental pollutants in these countries were recorded from May 1, 2022, to July 15, 2022.

### 2.1. Measurements of Air Pollutants and Monkeypox Cases

Data for the daily number of monkeypox cases were recorded from the official website of “Our World in Data, 2022” [16]. The daily concentrations of PM2.5, PM10, NO_2_, and O_3_ were obtained from the metrological website, “Real-Time Air Quality Index—AQI” [17]. The data was obtained starting from the first case of human monkeypox in these regions (May 1, 2022) to July 15, 2022.

The research team members visited the metrological website “Air Quality Index-AQI” and found day-to-day detailed information on air pollutants. AQI provided information about measurement protocols. Air pollutants were measured hourly over 24 hours each day. The AQI monitoring stations used high-tech laser particle sensors to measure real-time environmental pollution. One investigator obtained day-to-day information on air pollutants, PM 2.5, PM 10, NO_2_, and O_3_ concentrations, from the metrological websites (“Air Quality Index-AQI, 2021”). Also, another research team member checked the data on air pollutants daily concentration. The same research team members also recorded the human monkeypox daily cases from the official website of “Our World in Data.” However, for the confirmation of the data, another research team member rechecked the monkeypox cases in European countries.

### 2.2. Statistical Analysis

The results were analyzed using the “SPSS software version 22.0 for Microsoft windows.” The mean and SEM values were calculated using a paired sample *t* test. The linear and multiple regression analyses were performed to model the association of the air pollutants PM2.5, PM10, NO_2_, and O_3_ on the number of monkeypox day-to-day cases. The Spearman–Rho correlation was also used to assess the relationship, strength, and direction of the correlation between the pollutants and monkeypox cases. A *p* value <0.05 was considered statistically significant.

### 2.3. Ethical Statement

For this study, the data on the daily new cases of human monkeypox and particulate matter PM2.5, PM10, CO, NO_2_, and O_3_-related information were obtained from publicly available databases; hence, ethical approval was not required.

## 3. Results

The mean values for air pollutants PM2.5, PM10, NO_2_, and O_3_, and monkeypox cases in the United Kingdom and European countries are presented in Table 1. The highest levels of air pollutants and monkeypox cases were found in Spain. In Spain, the level of PM2.5 was (78.8 ± 3.3), PM10 (41.6 ± 2.0), NO_2_ (16.4 ± 0.5), and O_3_ (43.3 ± 1.1); and mean monkeypox cases were (101.3 ± 34.3).

The country with the second highest overall levels of air pollutants was the United Kingdom, with PM2.5 (68.0 ± 3.1), PM10 (27.8 ± 1.4), NO_2_ (39.6 ± 1.2), O_3_ (22.4 ± 0.7), and mean monkeypox cases were (64.0 ± 12.7). However, the lowest levels of air pollutants and lowest number of cases were found in Switzerland, where PM2.5 was (2.3 ± 0.1), PM10 was (15.2 ± 0.9), NO_2_ was (8.9 ± 0.3), and O_3_ was (34.0 ± 0.9), and monkeypox cases were (5.8 ± 1.0) (Table 1). The air pollutants and monkeypox cases data for other countries including Germany, France, the Netherlands, Italy, and Portugal, are also presented in Table 2.

Table 2 shows the combined mean values for air pollutants PM2.5, PM10, NO_2_, and O_3_, and monkeypox cases in the United Kingdom and the European continental countries (Figure 1). The highest overall concentration of all air pollutants, PM2.5, PM10, NO_2_, and O_3_ was found in Spain (45.0), which had the highest mean number of monkeypox cases (101.3). Furthermore, the overall combined lowest level of all air pollutants, PM2.5, PM10, NO_2_, and O_3_ was found in Switzerland (15.1) with the lowest mean number of monkeypox cases (5.8) during the study period (Table 2). Regarding other countries, the combined levels of air pollutants (PM2.5, PM10, NO_2_, and O_3_) were: the United Kingdom (38.7), France (35.7), Germany (21.8), and Netherlands (28.6), Portugal (22.1), and Italy (26.3). The number of monkeypox cases was in the United Kingdom (64), France (43.2), Germany (38.5), the Netherlands (32.3), Portugal (15.1), and Italy (13.5). The correlation coefficient was significant between the air pollutants and cases (*p* = 0.329).

A simple regression analysis was used to determine the effects of PM2.5, PM10, NO_2_, O_3_, and levels on monkeypox cases. The simple regression analysis results indicated that PM2.5 (*p* < 0.001), PM10 (*p* < 0.001), NO_2_(*p* < 0.05) and O_3_(*p* < 0.03) had a significant relationship with the number of daily monkeypox cases in the European countries (Table 3). Furthermore, multivariate regression analysis indicated that PM2.5 (*p* < 0.001) and O_3_(*p* < 0.002) each had a significant effect on the number of daily monkeypox cases. Therefore, in both simple and multiple regression analyses, the results indicated that both PM2.5 (*p* < 0.001), and O_3_(*p* < 0.002) had a significant effect on the number of daily monkeypox cases in European countries (Table 3).

The results further demonstrated that each 10-unit rise in PM-2.5, PM-10, NO_2_, and O_3_ levels was associated with a significantly increased number of monkeypox cases by 29.6%, 9.7%, 13%, and 80.6%, respectively.

In addition to simple and multiple linear regression analysis, Spearman's “Rho” correlation was executed to assess the strength and direction of the relationship between the air pollutant concentrations and the number of monkeypox cases. The Rho correlation analysis results are presented in Table 4. The results show that PM2.5, PM10, and NO_2_ had a significant linkage on the number of monkeypox cases PM2.5 (*p* < 0.001), PM10 (*p* < 0.001), and NO_2_(*p* < 0.001) (Table 4). However, O_3_ was borderline associated with an increased number of monkeypox cases (*p* = 0.06) (Table 4). The overall results reveal that the environmental pollutants PM2.5, PM10, and NO_2_ had a significant relationship with increased monkeypox cases (Table 4).

## 4. Discussion

The swift spread of human monkeypox viral infection has caused a challenging situation of global concern [10]. The present study explores the association of environmental pollutants PM 2.5, PM10, O_3_, and NO_2_ with the prevalence of monkeypox cases in European countries. We noted that PM2.5, PM10, O_3_, and NO_2_ were significantly associated with the number of monkeypox cases in eight European countries. This study also provides an understanding of the relationship between environmental pollutants and the occurrence of monkeypox cases.

The microorganisms, including bacteria, bacterial spores, viruses, and yeast are spread from infected patients to the inanimate environment, particularly to areas adjacent to patients and surfaces frequently touched by hands which we designate as “high-touch surfaces” [18]. Human MPXV transmission happens in two ways: either from animal to human or human to human. Monkeypox can spread from person to person through contact with an infected person's lesions or scabs that may be found on the skin or mucosal surfaces. Aerosol transmission has been demonstrated between animal populations [19]. Moreover, contact with an ill patient's respiratory droplets, secretions, lesion materials, body fluids, and polluted personal objects can contaminate the environment and result in the virus spreading among people [20].

The incubation period of human monkeypox diseases is about 7 days, ranging from 3–20 days [21]. The pox virus can remain active on linens, clothing, and environmental surfaces, particularly in dark, cool, and low-humidity environments. The virus can be contagious for about 15 days, and other closely related orthopoxviruses can persist in an environment similar to a household for weeks or months. The porous materials, bedding, and clothing may harbour the virus for longer periods than nonporous materials such as plastic, glass, and metal surfaces [22].

In the preprint literature (ahead of peer review) Susan et al. [23] reported that the monkeypox virus DNA lies on surfaces in hospitals and households. The monkeypox virus was found in air and dust samples that were collected during a bed linen change in rooms used to isolate monkeypox patients. The authors found widespread MPXV DNA contamination of the environment occupied by the infected and symptomatic individuals. Lin et al. [24] demonstrated that to control the spread of monkeypox disease in various regions or states, it is important to implement preventive and control measures for the isolation of patients and the appropriate disposal of pollutants. This study highlights the significance of pollutants which may spread diseases.

There is no available literature that can demonstrate the impact of particulate matter air pollutants on the incidence of monkeypox cases. To our knowledge, this is the first reported study to establish a relationship between air pollutants and the increasing number of monkeypox cases in European countries. It is worthwhile to consider the role of air pollutants in the spread of the SARS-CoV-2 virus during the COVID-19 pandemic. The SARS-CoV-2 virus is transmitted through various air pollutants. The medical literature indicates that environmental pollutants, including MP2.5, PM10, CO, NO_2_, and O_3_, can contribute to the spread of SARS-CoV-2 disease. Studies published from various regions of the globe including the United States of America [25], the United Kingdom [15], Italy [26], Saudi Arabia [27], and India [28], all have established a link between environmental pollution and an increased incidence of SARS-CoV-2.

The present study analysis demonstrates the strong influence of levels of particulate matter concentrations, positively associated with the number of daily cases of monkeypox in eight European countries. In the present study, it was identified that monkeypox cases were linked to some air pollutants (PM2.5, PM10, NO_2_, and O_3_) in eight different countries in Europe. The study findings favour the hypothesis that environmental pollutants are associated with increased daily cases of monkeypox in various countries.

### 4.1. Pathway between Air Pollutants and the Spread of Monkeypox Cases

This study has established plausible pathways to understand the relationship between increased air pollutants and increased cases of monkeypox in European countries. The monkeypox virus is inanimate but can be carried by air, dust, fine particles, ultrafine particles, and Earth substances. Environmental pollutants can carry the infected particles from surfaces and transport them over a long distance. Once the substances contaminated with monkeypox enter the environment, they can come into close contact with humans, enter the body, and infect people. The present study findings provide evidence that air pollutants can transport vectors for the monkeypox virus, promote viral entry into the body, and cause infection. These mechanisms are consistent with the hypothesis that air pollutants have resulted in an increased number of monkeypox cases in European countries.

## 5. Study Strengths and Limitations

This is the first novel study investigating the relationship between air pollutants and the number of monkeypox cases in European countries. The levels of air pollutants “PM2.5, PM10, O_3_, NO_2_, and monkeypox cases were temporally documented from the appearance of the first case of human monkeypox in eight European countries from May 1, 2022, to July 15, 2022. This study's findings established an association between environmental air pollutants and monkeypox cases. This study also has a few limitations. The first limitation of this study is that we were unable to collect the data for other pollutants, which may also be linked to affecting the dynamics of the monkeypox cases. Another limitation is that the number of monkeypox cases can fluctuate for reasons besides environmental pollutants. These reasons could include temperature, humidity, changes in societal patterns, gatherings, or noncompliance with preventive measures.

## 6. Conclusions

The levels of environmental pollutants, PM2.5, PM10, NO_2_, and O_3_ were significantly associated with an increased incidence of monkeypox cases in European countries. This is the first novel study that has established a linkage between air pollutants and monkeypox cases. These findings send a vital message to health authorities both at regional and global levels: take immediate measures to minimize environmental pollution to combat monkeypox disease.

## Figures and Tables

**Table 1:** The mean levels for air pollutants PM2.5, PM10, NO_2_, and O_3_, and monkeypox cases in European countries.

Countries	PM2.5 (ppm)	PM10 (ppm)	NO_2_ (DU)	O_3_ (ppm)	Monkeypox cases (*n*)
Spain	78.8 ± 3.3	41.6 ± 2.0	16.4 ± 0.5	43.3 ± 1.1	101.3 ± 34.3
United Kingdom	68.0 ± 3.1	27.8 ± 1.4	39.6 ± 1.2	22.4 ± 0.7	64.0 ± 12.7
Germany	32.2 ± 1.2	12.4 ± 0.5	1.9 ± 0.1	40.7 ± 1.1	38.5 ± 5.8
France	47.1 ± 1.3	35.5 ± 1.2	24.2 ± 0.9	35.9 ± 1.1	43.2 ± 11.5
Netherland	44.0 ± 1.2	22.4 ± 0.5	15.5 ± 0.6	32.4 ± 1.0	32.3 ± 7.8
Italy	53.6 ± 1.9	22.8 ± 1.1	11.4 ± 0.5	17.4 ± 0.4	13.5 ± 3.2
Portugal	29.9 ± 1.6	15.7 ± 0.8	11.4 ± 0.8	31.0 ± 0.9	15.1 ± 1.7
Switzerland	2.3 ± 0.1	15.2 ± 0.9	8.9 ± 0.3	34.0 ± 0.9	5.8 ± 1.0

Day-to-day data presented in Mean ± SEM from May 1, 2022, to July 15, 2022.

**Table 2:** The combined mean values for air pollutants PM2.5, PM10, NO_2_, O_3_, and monkeypox cases in European countries.

Countries	PM2.5, PM10, NO_2_, O_3_	Monkeypox cases	Correlation coefficient (*r*_*s*_)
Spain	45.0	101.3	0.329^*∗*^
United Kingdom	38.7	64	
France	35.7	43.2	
Germany	21.8	38.5	
Netherland	28.6	32.3	
Portugal	22.1	15.1	
Italy	26.3	13.5	
Switzerland	15.1	5.8	

**Table 3:** The linear and multiple regression analysis between PM-2.5, PM-10, NO_2_ and O_3_ and the number of monkeypox cases in European countries.

Simple regression				Multiple regression			
Variables	Β	*p* value	95% confidence interval		Β	*p* value	95% confidence interval	
			Lower	Upper			Lower	Upper
PM2.5 (ppm)	2.87	<0.001^*∗*^	1.94	4.26	2.96	<0.001^*∗*^	1.87	4.68
PM10 (ppm)	4.52	<0.001^*∗*^	2.10	10.21	0.97	0.962	0.29	3.22
NO_2_ (DU)	1.59	<0.05^*∗*^	1.00	2.54	1.30	0.420	0.69	2.44
O_3_ (DU)	3.78	<0.035^*∗*^	1.10	13.00	8.06	<0.002^*∗*^	2.19	29.65

^*∗*^Significant level.

**Table 4:** Spearman's Rho correlation between PM2.5, PM10, NO_2_, and O_3_ and the daily number of monkeypox cases in the United Kingdom and European countries.

Monkeypox cases	PM2.5 (ppm)	PM10 (ppm)	NO_2_ (DU)	O_3_ (ppm)
Spearman's rho (*r*_s_)	0.264^*∗*^	0.228^*∗*^	0.173^*∗*^	0.125
*p* value	<0.001	<0.001	<0.009	0.061

^*∗*^Significant level.

## Data Availability

Data may be provided on reasonable request to the corresponding author.
